# Supplementary material for: Supporting Smokers in Difficult Settings: Suggestions for Better Education and Counseling in Cancer Centers in Jordan
Source: Curr Oncol. 2022 Nov 30;29(12):9335–48. doi: 10.3390/curroncol29120732 (PMC9776699; doi:10.3390/curroncol29120732)
Supplement: Supplementary file 1 [file curroncol-29-00732-s001.zip › curroncol-1965716-supplementary.pdf]

Current Oncology

MDPI

Supplementals

Supporting Smokers in Difficult Settings: Suggestions for Better Education and Counseling in Cancer Centers in Jordan

Feras I. Hawari [1,2,†], Minas A. Abu Alhalawa [1,‡], Rasha H. Alshraiedeh [3,4,§], Ahmad M. Al Nawaiseh [4,5,‖], Alia Khamis [4,5], Yasmeen I. Dodin [1] and Nour A. Obeidat [1,*]

Table S1. Overview of measured items.

Item or construct	Detailed description	Operationalization for analyses
Tobacco use patterns	Pre-diagnosis and current patterns: daily cigarette consumption, time to first cigarette, days since last cigarette, number of quit attempts in the past, and smoking during treatment [1].	Cigarettes per day and number of days smoked in the past 30 days were used in the analysis.
Perceived nicotine dependence	"Would you consider yourself addicted to smoking?")[2].	Not included in current analysis.
Risk of relapse	Questions reflecting potential risk of relapse included: living with a smoker, in-door smoking allowed at home, smoking policy at work, past 30-day presence with smokers, extent to which subject is around smokers, cravings in the absence of a cigarette, and daily cigarettes smoked. Scores of 7 or higher imply relapse rates of approximately 75% [3].	A continuous variable was used. Relapse scores could range from 1 to 13.
Self-reported reasons for smoking	Open-ended item to capture self-reported reasons for smoking	Responses were qualitatively observed for insight.
Reasons for smoking and identity associated with smoking	Series of 14 items using a five-point Likert scale. The items reflect what others have documented regarding reasons for smoking: affiliative attachment, automaticity, loss of control, cognitive enhancement, cues or goads, taste, tolerance, weight control, tension reduction, pleasure, psychosocial reasons, sensorimotor reasons, boredom, identity linked to smoking [4-12].	Each item was dichotomized to express proportions of patients agreeing with statements (versus disagreeing or having no opinion).
Perceived effect of smoking on health and cause of cancer diagnosis	"To what extent do you think your smoking impacted your health?" and "what do you think contributed to your cancer diagnosis?"[13, 14].	Not included in current analysis.
Patient-reported receipt of information about the impact of smoking on cancer treatment	"What did your doctor or nurse inform you about smoking and its effect on your cancer treatment?"	Responses were qualitatively observed for insight.
Patient knowledge about the impact of smoking on various aspects of cancer treatment	Series of seven items (scaled as follows: from definitely increases, probably increases, no effect, probably decreases, definitely decreases, do not know) to gauge to what extent smoking could impact the risk of surgical complications, pain, stress, treatment efficacy, treatment side effects, relapse, and cancer survival [15].	Each item was dichotomized to express proportions of patients who thought smoking "definitely increases" or "definitely decreases" specific listed risks during cancer treatment (versus those who were not able to definitively

Item or construct	Detailed description	Operationalization for analyses
		identify risks of smoking in the context of cancer care).
Patient's response or emotional affect with regards to quitting	"How do you feel when you consider the idea of quitting"[16, 17].	Not included in current analysis.
Patient-reported facilitators and barriers to the process of smoking cessation	"What are the greatest challenges or barriers to your quitting?" and "What are the greatest facilitators that would/did help you quit?"[17].	Not included in current analysis.
Importance and confidence in quitting smoking	Importance and confidence were each measured on a ten-point scale [18].	A continuous variable was used for each. Values could range from 1 (not at all confident or not at all important) to 10 (extremely important or extremely confident).
Patient expectancies with regards to quitting	"What are the main positive and negative aspects of quitting that you would anticipate / that you experienced?"[9].	Not included in current analysis.
Reasons for quitting	Probed if the smoker quit after being diagnosed with cancer	Not included in current analysis.
How quitting happened	Among ex-smokers who quit after diagnosis, how the quit decision was made (suddenly or with prior planning) and how quitting proceeded (cold-turkey of gradual reduction).	Not included in current analysis.
Self-efficacy (maintenance and recovery self-efficacy)	Measured on a five-point Likert scale of perceived likelihood that the subject would remain committed to cessation given specific events (being around people who smoke, being stressed, when needing to concentrate, when craving a cigarette, and if a lapse occurred) [19, 20].	Not included in current analysis.
Perceived locus of control	Measured on a five-point Likert scale of agreement to the statements "by following a healthy lifestyle I can influence the course of my treatment", "I can influence the course of my treatment", and "becoming ill was a result of my lifestyle" (each measured using a five-point Likert scale of agreement) [21].	Not included in current analysis.
Availability of plans or tools to deal with difficult situations	Availability of plans or tools (if the patient had quit) [19].	Not included in current analysis.
Kind of change sought	Single item, measured only in those identifying as current smokers: choices were quitting, reduction, other change.	Not included in current analysis.
The use of action planning in those who had quit, and the likelihood of their use in those who were still smoking	Various specific action items were measured using either a yes/no/don't remember response (for those who had already quit), or a five-point Likert scale of perceived likelihood that the subject would employ such techniques were they to quit (for those currently smoking). These included getting rid of tobacco products and ashtrays, setting a quit date,	Not included in current analysis.

Item or construct	Detailed description	Operationalization for analyses
	thinking of ways to control weight, breaking habits of smoking in specific places or times, informing people about quitting intention, trying to find aids to quit, avoiding smoking triggers, finding a quit buddy, and asking for support from family and friends [22]. We also included a statement "If smoking out of control, I do something".	
Employment of self-change strategies	Items measured the extent to which (frequency on a give-point Likert scale) subjects "think about benefits of having quit", "avoid smoking areas" and "avoid situations that make me smoke". Among those who currently smoked, additional items included "tell myself to stop", "wait as long as I can to smoke", and "think about harms of my smoking". Among those who had quit, additional items included "feel accomplishment due to stopping smoking" and "promise self not to return to smoking again" [23].	Not included in current analysis.
Procedural knowledge	Procedural knowledge about the cessation process. Items ("quitting smoking needs skills", "the steps to quit are clear", "I have the resources I need to quit") were measured using a five-point Likert scale of extent of agreement with the statement [24, 25] .	Not included in current analysis.
Perceived difficulty in quitting and need for help	Extent of agreement (on a five-point Likert scale) with "quitting smoking is easy" and "I can quit on my own without any help"	Not included in current analysis.
Smoking cessation fatigue	Measured only in those identifying as current smokers, the extent to which (on a five-point Likert scale of agreement) current smokers felt fatigued by cessation thoughts such as being drained from trying to quit, feeling that trying to quit was pointless, feeling discouraged or overwhelmed when thinking of quitting; and, conversely, the extent to which current smokers felt optimistic about quitting or felt like quitting was nearer at hand and worth it, or that smokers understood what works towards their quitting [26].	Not included in current analysis.
Norms	Extent of agreement (on a five-point Likert scale) with "my family wants me to quit" and "most people important to me want me to quit"	Not included in current analysis.
Perceptions regarding smoking cessation services within Center	Various statements specific to the process of care at the center and tapping into previous results obtained from providers at the center with regards to patient-related barriers to smoking cessation [27]. These included statements about perceived support for smoking cessation support from providers in the center, attitudes towards providers initiating the subject of smoking cessation, the extent to which patients perceived smoking cessation to be a responsibility they assume, the general perception	Not included in current analysis.

Item or construct	Detailed description	Operationalization for analyses
	of the environment at the center as a site conducive to smoking cessation, receipt of smoking cessation advice, and referral to the smoking cessation clinic.	

References

1. Land, S.R.; Toll, B.A.; Moinpour, C.M.; Mitchell, S.A.; Ostroff, J.S.; Hatsukami, D.K.; Duffy, S.A.; Gritz, E.R.; Rigotti, N.A.; Brandon, T.H. et al. Research Priorities, Measures, and Recommendations for Assessment of Tobacco Use in Clinical Cancer Research. Clinical cancer research. *Clin Cancer Res*. **2016**, *22(8)*, 1907-1913.
2. Berg, C.J.; Schauer, G.L.; Buchanan, T.S.; Sterling, K.; DeSisto C.; Pinsker, E.A.; Ahluwalia, J.S. Perceptions of addiction, attempts to quit, and successful quitting in nondaily and daily smokers. *Psychol. Addict. Behav.* **2013**, *27(4)*, 1059-1067.
3. Bolt, D.M.; Piper, M.E.; McCarthy, D.E.; Japuntich, S.J.; Fiore, M.C.; Smith, S.S.; Baker, T.B.: The Wisconsin Predicting Patients' Relapse questionnaire. *Nicotine Tob. Res.* **2009**, *11(5)*, 481-492.
4. Smith, S.S.; Piper, M.E.; Bolt, D.M.; Fiore, M.C.; Wetter, D.W.; Cinciripini, P.M.; Baker, T.B. Development of the Brief Wisconsin Inventory of Smoking Dependence Motives. *Nicotine Tob. Res.* **2010**, *12(5)*, 489-499.
5. Berlin, I.; Singleton, E.G.; Pedarriosse, A.-M.; Lancrenon, S.; Rames, A.; Aubin, H.-J.; Niaura, R. The Modified Reasons for Smoking Scale: factorial structure, gender effects and relationship with nicotine dependence and smoking cessation in French smokers*. *Addiction* **2003**, *98*, 1575–1583, https://doi.org/10.1046/j.1360-0443.2003.00523.x.
6. De Wilde, K.S.; Tency, I.; Boudrez, H.; Temmerman, M.; Maes, L.; Clays, E. The Modified Reasons for Smoking Scale: factorial structure, validity and reliability in pregnant smokers. *J. Eval. Clin. Pract.* **2016**, *22(3)*, 403-410.
7. Souza, E.S.; Crippa, J.A.; Pasian, S.R.; Martinez, J.A. University of Sao Paulo Reasons for Smoking Scale: a new tool for the evaluation of smoking motivation. *J. Bras. Pneumol.* **2010**, *36(6)*, 768-778.
8. Tate, J.C.; Pomerleau, C.S.; Pomerleau, O.F. Pharmacological and non-pharmacological smoking motives: a replication and extension. *Addiction.* **1994**, *89(3)*, 321-330.
9. Hendricks, P.S.; Wood, S.B.; Baker, M.R.; Delucchi, K.L.; Hall, S.M. The Smoking Abstinence Questionnaire: measurement of smokers' abstinence-related expectancies. *Addiction.* **2011**, *106(4)*, 716-728.
10. Rash, C.J.; Copeland, A.L. The Brief Smoking Consequences Questionnaire-Adult (BSCQ-A): development of a short form of the SCQ-A. Nicotine & tobacco research *Nicotine Tob. Res.* **2008**, *10(11)*, 1633-1643.
11. Dupont, P.; Tack, V.; Blecha, L.; Reynaud, M.; Benyamina, A.; Amirouche, A.; Aubin, H.J. Smoker's identity scale: Measuring identity in tobacco dependence and its relationship with confidence in quitting. *Am. J. Addict*. **2015**, *24(7)*, 607-612.
12. Farrimond, H.; Joffe, H.; Stenner, P. A Q-methodological study of smoking identities. *Psychol. Health.***2010**, *25(8)*, 979-998.
13. Carter-Harris, L.; Ceppa, D.P.; Hanna, N.; Rawl, S.M. Lung cancer screening: what do long-term smokers know and believe? Health expectations *Health Expect.***2017**, *20(1)*, 59-68.
14. Bottorff, J.L.; Robinson, C.A.; Sullivan, K.M.; Smith, M.L. Continued family smoking after lung cancer diagnosis: the patient's perspective. *Oncol. Nurs. Forum..* **2009**, *36(3)*, E126-132.
15. Obeidat, N.A.; Hawari, F.I.; Amarin, R.; Altamimi, B.A.; Ghonimat, I.M. Educational Needs of Oncology Practitioners in a Regional Cancer Center in the Middle East-Improving the Content of Smoking Cessation Training Programs. Journal of cancer education *J. Cancer Educ.* 2016.
16. Watson, D.; Clark, L.A.: The PANAS-X - Manual for the Positive and Negative Affect Schedule - Expanded Form. The University of Iowa. https://ir.uiowa.edu/cgi/viewcontent.cgi?article=1011&context=psychology_pubs/. 1994.
17. van Eerd, E.A.; Risor, M.B.; van Rossem, C.R.; van Schayck, O.C.; Kotz, D. Experiences of tobacco smoking and quitting in smokers with and without chronic obstructive pulmonary disease-a qualitative analysis. *BMC Fam. Pract*. 2015, *16*, 164.

18. Miller, W.R.; and Rollnick, S. Motivational Interviewing: Helping People Change. 3rd ed. New York, NY: Guilford Press, 2013.
19. Elfeddali, I.; Bolman, C.; Candel, M.J.J.M.; Wiers, R.W.; De Vries, H. The role of self-efficacy, recovery self-efficacy, and preparatory planning in predicting short-term smoking relapse. *Br. J. Heal. Psychol.* **2011**, *17*, 185–201, https://doi.org/10.1111/j.2044-8287.2011.02032.x.
20. Schwarzer, R.; Lippke, S.; Luszczynska, A. Mechanisms of health behavior change in persons with chronic illness or disability: The Health Action Process Approach (HAPA).. *Rehabilitation Psychol.* **2011**, *56*, 161–170, https://doi.org/10.1037/a0024509.
21. Cousson-Gelie, F.; Irachabal, S.; Bruchon-Schweitzer, M.; Dilhuydy, J.M.; Lakdja, F. Dimensions of cancer locus of control scale as predictors of psychological adjustment and survival in breast cancer patients. *Psychol. Rep.* **2005**, *97(3)*, 699-711.
22. de Vries, H.; Eggers, S.M.; Bolman, C. The role of action planning and plan enactment for smoking cessation. *BMC Public Health* 2013, *13*, 393.
23. Etter, J.F.; Bergman, M.M.; Perneger, T.V. On quitting smoking: development of two scales measuring the use of self-change strategies in current and former smokers (SCS-CS and SCS-FS). *Addict. Behav.* **2000**, *25(4)*, 523-538.
24. Cane, J.; O'Connor, D.; Michie, S. Validation of the theoretical domains framework for use in behaviour change and implementation research. *Implementation science* : IS **2012**, *7*, 37.
25. Michie, S.; Johnston, M.; Abraham, C.; Lawton, R.; Parker, D.; Walker, A. Making psychological theory useful for implementing evidence based practice: a consensus approach. *Qual. Saf. Health Care.* **2005**, *14(1)*, 26-33.
26. Mathew, A.R.; Heckman, B.W.; Meier, E.; Carpenter, M.J. Development and initial validation of a cessation fatigue scale. *Drug Alcohol Depend*. **2017**, *176*, 102-108.
27. Obeidat, N.A.; Ayub, H.S.; Amarin, R.; Aburajab Altamimi, B.; Ghonimat, I.; Abughosh, S.; Hawari, F.I. Smoking Cessation Support Among Oncology Practitioners in a Regional Cancer Center in the Middle East-Improving a Critical Service for Cancer Care. *Oncologist* **2016**, *21(4)*, 503-505.
